# Designing clinical and genetic guidelines of colorectal cancer screening as an effective roadmap for risk management

**Published:** 2016-12

**Authors:** Mohammad Reza Zali, Reza Safdari, Elham Maserat, Hamid Asadzadeh Aghdaei

**Affiliations:** 1 *Gastroenterology and Liver Diseases Research Center, Research Institute for Gastroenterology and Liver Diseases, Shahid Beheshti University of Medical Sciences, Tehran, Iran*; 2 *Allied Medical Sciences School, Tehran University of Medical Sciences, Tehran, Iran*; 3 *Management and Medical Informatics School, Tabriz University of Medical Sciences, Tabriz, Iran*

**Keywords:** Colorectal Cancer, Screening, Guideline

## Abstract

**Aim:**

We aimed to present clinical and genetic guidelines of colorectal cancer screening for risk assessment of populations at risk.

**Background:**

National guidelines can be used as a guide for choosing the method of screening for each individual. These guidelines facilitate decision making and support the delivery of cancer screening service.

**Methods:**

In the first step, a comparative study was performed by using secondary data extracted from the literature review. Three countries (Canada, Australia and United States) were selected from 25 countries that are member in the International Cancer Screening Network (ICSN). The second step of study was qualitative survey. The study was based on the grounded theory approach. Study tool was semi-structured interview. Interviewing involves asking questions and getting answers from participants. 22 expert’s perspectives about guidelines of colorectal cancer screening were surveyed.

**Results:**

Screening program of selected countries was compared. Countries were surveyed by number of risk groups and subgroups, criteria for risk assessment, beginning age, recommendations, screening approaches and intervals. Australia and United States have three risk groups and Canada has two risk groups. Four risk groups were defined in the national guideline, including high risk, increased risk, average and low risk group. The high risk group comprises of 8 subgroups, increased risk group comprises of 3 subgroups and average risk group contain 4 subgroups. Approved clinical criteria for hereditary syndromes and the roadmap of genetic and pathologic survey were designed.

**Conclusions:**

Guidelines and pathways have a vital role in the quality improvement of CRC screening program. National guidelines were refined according to the environmental and genetic criteria of colorectal cancer in Iran. These guidelines provide evidence-based recommendations by risk groups. National pathways as a risk assessment tool can evaluate and improve the processes and outcomes of cancer screening in practice. One of the suggestions for future research is the designing expert system for real-time decision making during a clinical interaction.

## Introduction

Colorectalcancer(CRC)incidenceandmortalityarereduced with regular screening (1-4). Guidelines and pathways can be used as a tool for choosing the best screening approach (5). Clinical practice guideline introduces as a strategy to reduce variability in screening, improve quality, measure outcome, and manage costs. The clinical practitioners have endorsed the use of clinical guidelines as the scientific evidence and expert opinions to achieve consensus about best practice and most efficient way to reduce costs. However, Also guidelines are the strategy of our federal agencies to reduce variability in care, improve quality, measure outcomes, and reduce costs (6). The purpose of this guideline is to present the available evidence for evaluation of all clinical situations (7). These guidelines provide health care providers with responsible recommendations on recall, maintenance, and track for patients and relatives (8). These pathways represent evidence-based recommendations on best practices in disease management to provide the highest level of cancer care (9). AGA presents a broad portfolio of guidelines, decision support tools and standards to support high-quality patient care for colorectal cancer screening. These guidelines define and early detect key populations to include both vulnerable and most-at-risk populations (10). However, studies show that multidimensional guidelines can make preventive care services (11). These comprehensive guidelines improve the quality of care and can make clear communication among clinicians (12).

Decision strategiesstrategies for screening have become increasingly complex in recent decades. Screening pathway provide more and new options for cancer management (13). Special emphasis on the designing of user-friendly referral clinical guidelines, the integrated incorporation of computer- based recall to facilitate risk assessment recommendation is required (14-18). Key statistics for colorectal cancer have shown several risk factors that might increase a person’s chance of developing polyps or CRC (19). The survey of genetic variants and environmental risk may permit individualized risk stratification for CRC as part of routine care. The quality of risk stratification tools has a key role in optimization of screening plan (20). Clinicians and other experts involved in the CRC screening state the need for clear guidance on the risk classification and referral process in clinical practice settings (17, 21). We present national risk assessment guidelines for screening plan. Four risk groups were defined, including high risk group, increased group, average and low risk group. These evidence-based clinicalclinical recommendations identify roadmap of detection and removal of adenomatous polyps. CRC largely can be prevented by the early detection of polyps. National guidelines can play an integral role in CRC control by detecting those individuals whose behavior, environment or family history place them at high risk populations. The objective was to provide guidelines for colorectal cancer screening with an emphasis on the care of patients who are at risk for colorectal cancer.

## Materials and Methods

In the first stage, a comparative study was conducted by using secondary data extracted from the literature review. Three countries were selected from 25 countries that are member in the international Cancer Screening Network (ICSN). ICSN is a voluntary consortium of countries that have active population- based cancer screening programs and continuing efforts to evaluate and optimize the cancer screening plan in clinical setting (22). Selected countries (Canada, Australia and United States) had clear and comprehensive screening plan by risk groups.

The second stage of study was qualitative survey. The study was based on the grounded theory approach (23). Study tool was semi-structured interview. Interviewing involves asking questions and getting answers from participants. 22 expert’ perspectives about guidelines of colorectal cancer screening were surveyed. Participants were informed and had experience about screening program. This sample provided sufficient numbers to ensure exploration of the fields, and data saturation was reached by the final interviews. Participants were asked about number of risk groups and subgroups, criteria for risk assessment, beginning age, recommendations, screening approaches and intervals. All interviews were fully transcribed and coded and analyzed by two researchers. We explained the study and obtained initial consent for further contact from participants. Interviewing involves asking questions and getting answers from participants. We explained the purpose of the study and confidentially of information for participation. Also we asked for consent to audio-record the interviews. Data was gathered by two researchers between January and April 2015. Finally, coded data was organized. All of guidelines were approved by clinical and genetic experts.

## Results

Screening program of selected countries (Canada, Australia and United States) was illustrated in table 1. The beginning age for colonoscopic screening was 50 in each three countries (24-26). Australia and United States have three risk groups and Canada has two risk groups.

**Figure 1:**
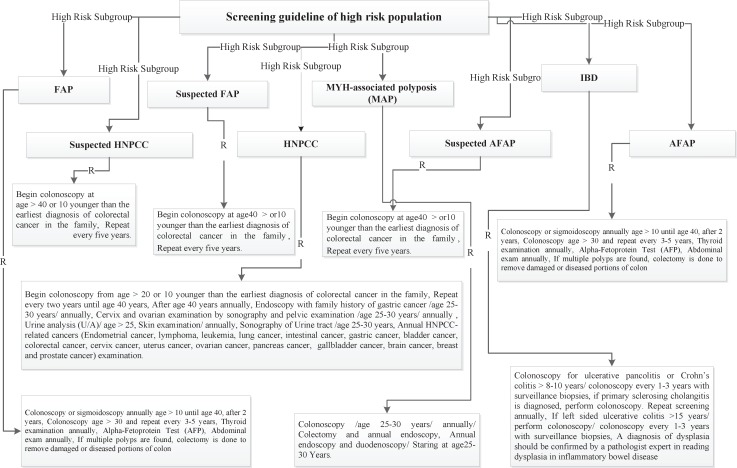
Screening guideline of high risk population

**Figure 2:**
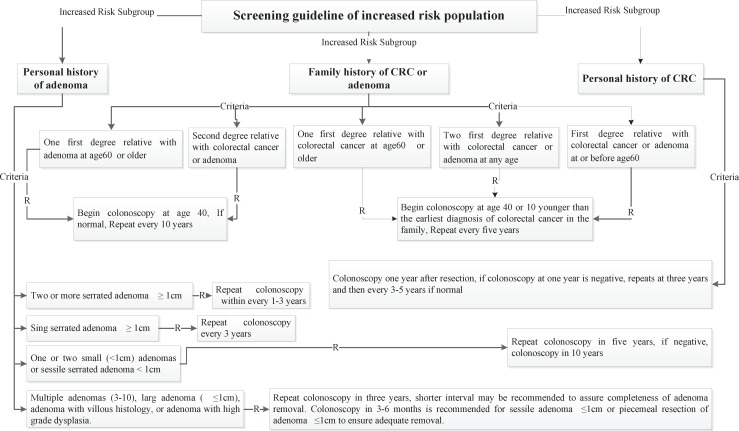
Screening guideline of increased risk population

**Figure 3:**
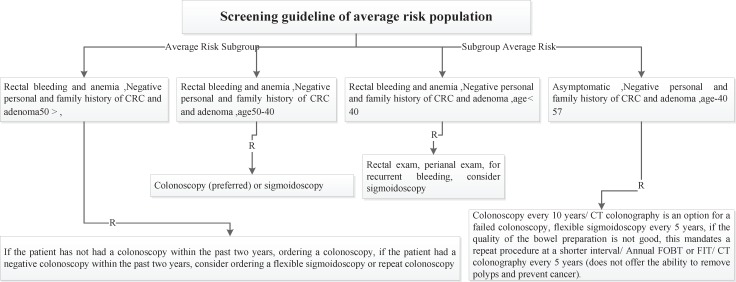
Screening guideline of average risk population

**Figure 4:**
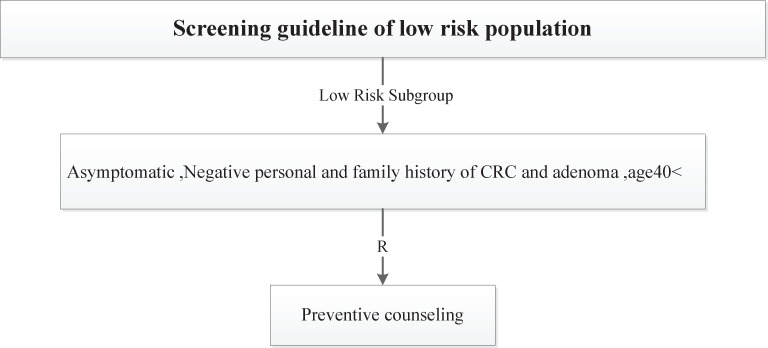
Screening guideline of low risk population

**Figure 5:**
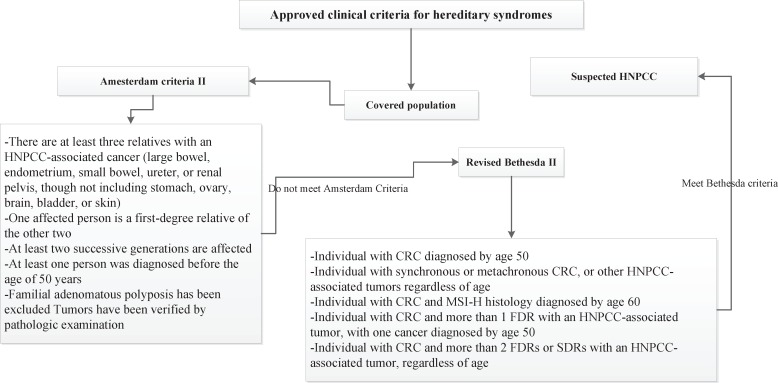
Approved clinical criteria for hereditary syndromes

**Figure 6:**
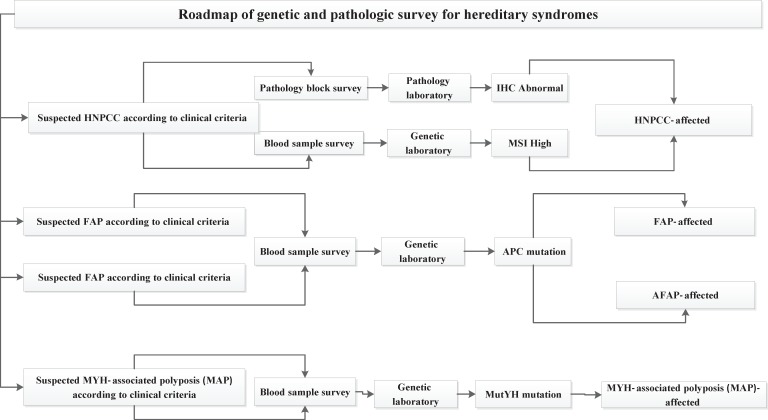
Roadmap of genetic and pathologic survey for hereditary syndromes

Four risk groups were defined in the national guideline, including high risk, increased risk, average and low risk group. The high risk group comprises of 8 subgroups (FAP, AFAP, Suspected FAP, Suspected FAP, HNPCC, Suspected HNPCC, MYH, IBD). Guideline of high risk group was illustrated in figure 1. The increased risk group comprises of 3 subgroups (Personal history of adenoma, Personal history of CRC, Family history of CRC and adenoma). Guideline of increased risk group was illustrated in figure 2. The group of family history of CRC and adenoma contain 5 groups, include one first degree relative with colorectal cancer or adenoma at or before age 60 years, two first degree relative with colorectal cancer or adenoma at any age, one first degree relative with colorectal cancer at age 60 or older, second degree relative with colorectal cancer or adenoma and one first degree relative with adenoma at age 60 years or older. The average risk group comprises of 4 subgroups (Asymptomatic, Negative personal and family history of CRC and adenoma, age 40-57 years/ Rectal bleeding and anemia, Negative personal and family history of CRC and adenoma, age < 40 years/ Rectal bleeding and anemia, Negative personal and family history of CRC and adenoma, age 40-50 years/ Rectal bleeding and anemia, Negative personal and family history of CRC and adenoma, age > 50 years). Guideline of average risk group was illustrated in figure 3. The low risk group comprises of asymptomatic, negative personal and family history of CRC and adenoma, age < 40 years. Guideline of low risk group was illustrated in figure 4. Approved clinical criteria for hereditary syndromes were demonstrated in figure 5. Roadmap of genetic and pathologic survey was shown in figure 6.

**Table 1:** Comparative study of screening guidelines in selected countries

Country		Number of risk group		Criteria	Subgroup	Screening method			Age of screening	Interval screening	
Canada		2 groups		Symptomatic/ Asymptomatic regardless of age but positive family history	HNPCC	Colonoscopy			Begin colonoscopy at age 20 or 10 younger than the earliest diagnosis of colorectal cancer in the family	1-2 years	
					FAP	Sigmoidoscopy			Begin at age 10-12 years	Annually	
					AFAP	Colonoscopy			Begin at age 16-18 years	Annually	
					One first degree relatives with cancer or adenomatous polyp at age >60 or Two or more first degree relatives with polyp or colon cancer at any age	Colonoscopy			Begin colonoscopy at age 40 or 10 younger than the earliest diagnosis of colorectal cancer in the family	Every five years	
					One first degree relatives with cancer or adenomatous polyp at age <60 or Two or more second degree relatives with polyp or colon cancer at any age	Colonoscopy			Begin at age 40 years	-	
					One second degree relative or third degree relative affected	Colonoscopy			Begin at age 50 years	-	
					Polyps found at colonoscopy/ 1-2 tubular adenomas<1cm	Colonoscopy			-	In five years	
					Polyps found at colonoscopy/>2 adenomas	Colonoscopy			-	In 3 years	
					Ulcerative colitis or Crohn’s colitis	Colonoscopy			-	At age 8-10 years	
Australia		3 groups		Symptoms of CRC	Abdominal pain/Unexplained tiredness/weight loss/ Lump/mass in tummy (abdomen)/Rectal bleeding/A persistent (beyond 2 week) change in bowel habit	Colonoscopy		-		Within 30 day	
				Personal history of CRC and adenoma	Bowel cancer, Polyps. IBD such as Ulcerative colitis or Crohn’s colitis	Colonoscopy		-		Within 30 day	
				Family history of CRC and adenoma	One first degree and ≥ 2 first or second degree relatives on the same side of the family OR One first degree and ≥ 1 first or second degree relatives on the same side of the family diagnosed with: - multiple bowel cancers in one person - bowel cancer < 50 years - other HPNCC related cancers	Colonoscopy		from age 25 or 5 years earlier than the youngest relative diagnosed, whichever comes first.		every 1-2 years	
					Relatives diagnosed with FAP	flexible sigmoidoscopy or colonoscopy		From age 12-15 or from diagnosis.		Annually	
					Relatives diagnosed with Lynch Syndrome (HNPCC)	Colonoscopy		from age 25 or 5 years earlier than the youngest relative diagnosed, whichever comes first.		every 1-2 years	
					One first degree relative diagnosed with bowel cancer < 55 years OR Two first or second degree relatives on the same side of the family diagnosed with bowel cancer at any age.	Colonoscopy		from age 50 every 5 years or 10 years earlier than the youngest relative diagnosed with bowel cancer, whichever comes first.		every 5 years	
					One first degree relative diagnosed with bowel cancer ≥ 55 years	FOBT		-		-	
				High risk	HNPCC	Colonoscopy		Age 20 to 25 years, or 10 years before the youngest case in the immediate family		every 1 to 2 years	
					FAP	flexible sigmoidoscopy or colonoscopy		Age 10 to 12		Annually	
					IBD	Colonoscopy		8 years after the onset of pancolitis (involvement of entire large intestine), or 12-15 years after the onset of left-sided colitis		every 1 to 2 years	
					People with small rectal hyperplastic polyps	Colonoscopy		Starting at age 50		every 10 years	
					People with 1 or 2 small (less than 1 cm) tubular adenomas with low-grade dysplasia	Colonoscopy		5 to 10 years after the polyps are removed		Time between tests should be based on other factors such as prior colonoscopy findings, family history, and patient and doctor preferences.	

USA		3 groups		Increased risk (Personal history polyp and CRC/ family history polyp and CRC)		
						People with 3 to 10 adenomas, or a large (at least 1 cm) adenoma, or any adenomas with high-grade dysplasia or villous features	Colonoscopy	3 years after the polyps are removed			every 5 years
						People with more than 10 adenomas on a single exam	Colonoscopy	3 years after the polyps are removed			every 3 years
						People with sessile adenomas that are removed in pieces	Colonoscopy	2 to 6 months after adenoma removal			doctor’s judgment
						People diagnosed with colon or rectal cancer	Colonoscopy	At time of colorectal surgery, or can be 3 to 6 months later if person doesn’t have cancer spread that can’t be removed			If the tumor presses on the colon/rectum and prevents colonoscopy, CT colonoscopy (with IV contrast) or DCBE may be done to look at the rest of the colon.
						People who have had colon or rectal cancer removed by surgery	Colonoscopy	Colonoscopy one year after resection,			if colonoscopy at one year is negative, repeats at three years and then every 3-5 years if normal
						Colorectal cancer or adenomatous polyps in any first- degree relative before age 60, or in 2 or more first-degree relatives at any age (if not a hereditary syndrome).	Colonoscopy	Age 40, or 10 years before the youngest case in the immediate family, whichever is earlier			every 5 years
						Colorectal cancer or adenomatous polyps in any first- degree relative aged 60 or older, or in at least 2 second- degree relatives at any age	Colonoscopy	Age 40 years			every 10 years
				Average risk		Asymptomatic, Negative personal and family history of CRC and adenoma, age 40-57	Colonoscopy	-			every 10 years

## Discussion

We presented a comparative review of the current literature on screening strategies. This review was basedbased on designing of clinical and genetic guidelines. In this study we defined four risk groups. Screening of HNPCC as a high risk group has relied on analysis of the family history and other clinic- pathological criteria, such as the Amsterdam and Bethesda criteria (27). Individuals with suspected HNPCC were detected by clinical criteria in the first step. Approved guidelines contain two clinical criteria, including Amsterdam and Bethesda criteria. Predictive examination of HNPCC is conducted within genetic counseling protocol (28). National guidelines covered roadmap of counseling protocol. Health care professionals are involved in CRC screening program suggested indicators for quality improvementimprovement of the pilot CRC plans (29). This survey details all of criteria and indicators for risk assessment. Emphasis on the golden standards is significant for implementing of screening plans (30). The outcomes of population- based screening programs have shown colonoscopy is a prevention tool against colorectal cancer (31). We present colonoscopy as a prevention tool especially among high risk populations. In addition, Screening for CRC is a complex process (32, 33). User-friendly referral clinical guidelines facilitate this complex process by recognized recommendations (14-18). This document includes comprehensive and clear guidelines for risk stratification that help healthcare professionals to better detect the colorectal cancer at an early stage. Studies show that adenoma detection to reduce CRC rates (34). Formulated national guidelines were classify polyps and recommended appropriate screening methods for investigating of polyps. Individuals with personal and family history of adenoma were classified by guidelines. According to our current studies, 75% of diagnosed CRC are sporadic (35). In this survey, individuals with familial/inherited and sporadic colon cancer were classified separately. Screening approaches were defined for sporadic colon cancer and demonstrated in guideline structure. International guidelines suggest the idea of colonoscopy surveillance after detection and removal of polyps (36). National guidelines interpreted colonoscopy interval and other criteria after removal of polyps in covered populations. According to these studies, it is accepted that organized guidelines facilitate colorectal cancer screening.

In summary, clinical and genetic guidelines have a key role in quality improvement of CRC screening. National guidelines were refined according to the environmental and genetic criteria of colorectal cancer in Iran. These guidelines provide evidence- based recommendations by risk groups. National pathways as a risk assessment tool can evaluate and improve the processes and outcomes from cancer screening in practice. Screening guidelines need to be integrated with clinical process for providing suitable patient-specific advice. Considering to results of this study is useful for optimaloptimal implementing of a risk assessment system. As a conclusion, it is recommended to consider the necessity of integration standards for risk assessment.

One of the suggestions for future research is to design an intelligent system for real-time decision making during a clinical interaction. Electronic system identified gaps between the existing guidelines functionality and the needs of health care providers of the screening program.
